# Application of Deep Learning and Intelligent Sensing Analysis in Smart Home

**DOI:** 10.3390/s24030953

**Published:** 2024-02-01

**Authors:** Yi Lu, Lejia Zhou, Aili Zhang, Siyu Zha, Xiaojie Zhuo, Sen Ge

**Affiliations:** 1 College of Art and Design, Beijing University of Technology, Beijing 100124, China; zhoulejia@emails.bjut.edu.cn (L.Z.); zalchina@126.com (A.Z.); gesen@emails.bjut.edu.cn (S.G.); 2 The Future Laboratory, Tsinghua University, Beijing 100084, China; zhasiyu@mail.tsinghua.edu.cn; 3 Faculty of Innovation and Design, City University of Macau, Macau 999078, China; u22092110437@cityu.edu.mo

**Keywords:** smart home, optical sensors, user behavior recognition, deep learning, cloud computing, intelligent sensing

## Abstract

Deep learning technology can improve sensing efficiency and has the ability to discover potential patterns in data; the efficiency of user behavior recognition in the field of smart homes has been further improved, making the recognition process more intelligent and humanized. This paper analyzes the optical sensors commonly used in smart homes and their working principles through case studies and explores the technical framework of user behavior recognition based on optical sensors. At the same time, CiteSpace (Basic version 6.2.R6) software is used to visualize and analyze the related literature, elaborate the main research hotspots and evolutionary changes of optical sensor-based smart home user behavior recognition, and summarize the future research trends. Finally, fully utilizing the advantages of cloud computing technology, such as scalability and on-demand services, combining typical life situations and the requirements of smart home users, a smart home data collection and processing technology framework based on elderly fall monitoring scenarios is designed. Based on the comprehensive research results, the application and positive impact of optical sensors in smart home user behavior recognition were analyzed, and inspiration was provided for future smart home user experience research.

## 1. Introduction

In recent years, the rapid development of the IoT industry and mobile internet technology has promoted the construction of cloud computing platforms and provided the smart home system with the ability to work together across platforms and devices, which effectively improves the response speed and processing capability of user behavior recognition. In smart homes, optical sensors, as one of the main information perception tools, can play an important role in behavior recognition, environmental monitoring, material analysis, etc. With the advantages of being non-invasive, having less restriction of behavior detection and being low cost, optical sensors can obtain user behavior information by sensing changes in different light and provide real-time user behavior data, which are a hot topic of concern for scholars in various fields [1,2,3]. However, affected by complex home environments such as lighting conditions, the number of users, the range and amplitude of activities, intelligent terminals have to process more and more information. As an emerging model of resource usage and delivery, cloud computing technology can provide powerful computing power, sufficient network resources and huge storage space in the cloud. Based on cloud computing technology, the study combines the context-aware technology used to explore the user needs of the smart home, and reasonably applies various types of optical sensors for user behavior recognition, which helps to improve the efficiency of data use while reducing the maintenance cost of the product, construct a good interaction process and user experience, and realize the diversified development of the smart home.

This article focuses on the application of optical sensors in user behavior recognition within smart homes, using cloud computing technology. It systematically reviews conference and journal papers from the Web of Science and Scopus databases that relate to this application and conducts a visual analysis of the literature using CiteSpace (Basic version 6.2.R6) software. The study examines the working principles and case studies of user behavior recognition using optical sensors, analyzing its technical framework. It delves deeply into the evolution and cutting-edge themes of this field through timeline maps and identifies research hotspots and future trends through the analysis of keyword co-occurrence and clustering maps. Integrating these findings, the paper explores the advantages and applications of optical sensor-based user behavior recognition in smart homes, particularly in scenarios like elderly fall prevention, grounded in cloud computing technology. Adhering to a user-centered design principle, it aims to support research in the smart home domain and inspire innovative design within the field.

## 2. Methodology

The analytical tool used in this study is CiteSpace, an information visualization software developed using Java. It primarily employs co-citation analysis and the PathFinder network algorithm, among other techniques, to quantitatively analyze the literature and create visual maps [4]. These maps reveal key paths and knowledge turning points in the evolution of academic fields, aiding researchers in quickly identifying and understanding the hotspots and frontier trends in relevant scientific areas.

In selecting the literature, the study strives to meet standards of high internationalization and timeliness, focusing specifically on the field of optical sensor applications in smart home user behavior recognition, which holds targeted relevance and core influence. Therefore, the literature search primarily sourced English journal articles and conference papers from the Web of Science and Scopus databases, covering the period from 2013 to 2022. The extracted data comprehensively include details such as titles, authors, keywords, abstracts, publication dates, citation counts, and references.

This paper conducts literature searches based on thematic words, including search titles, abstracts, author keywords, and additional keywords, to find papers highly relevant to research on optical sensor-based user behavior recognition in smart homes. The search criteria for thematic words are as follows: (1) With smart homes as the primary research context and considering their characteristics and usage environments, the first-level search theme revolves around three categories—“intelligence”, “home”, and “product”. The related thematic word collection includes “smart, intelligen*, automat*”; “home*, household, house, domestic, room, kitchen, toilet, basement, garden”; “product, supplies, item, appliance”, searching for papers containing two or more of these thematic words. (2) The main subject of this study is optical sensors, so the second-level search theme is related to “optical sensors”, with a thematic word collection of “photoelectric sensor, optical sensor, image sensor, IR sensor, laser sensor, light sensor, UV sensor, photoelectric transducer, optical transducer, image transducer, IR transducer, laser transducer, light transducer, UV transducer”. (3) Finally, literature related to user behavior recognition is searched with “recognition” as the third-level search theme, with the main thematic word collection including “recognition, recognize, identification, identity”.

After filtering through these three levels of search themes in the Web of Science and Scopus databases, each title, abstract, and keyword was manually reviewed to exclude papers with low relevance to optical sensor-based smart home user behavior recognition research (including smart products in other fields, applications of optical sensors in other fields, etc.). Ultimately, 51 papers of high relevance were selected as the core data source for case analysis and visualization mapping. These papers were used to summarize the working principles and review tables in the following sections, and to create timeline maps and keyword co-occurrence and clustering maps.

## 3. Application of Optical Sensors in Smart Home User Behavior Recognition

### 3.1. Optical Sensor Types and User Behavior Recognition Working Principle

The optical sensor takes the light signal as the sensing information carrier and uses a detector to convert the light signal into an electrical signal for data processing. In the process of smart home user behavior recognition, by measuring and monitoring the light signal reflected, transmitted, and scattered by external physical parameters, and analyzing the changes in the characteristic parameters of the light signal (such as wavelength, intensity, phase, polarization, etc.) caused by the user or the environment, etc., optical sensors can achieve functions such as temperature measurement, distance measurement, speed measurement, 3D imaging, and positioning [5]. As shown in Table 1, the placement of light-source transmitters and receivers in smart homes varies for different application scenarios and optical sensors [6,7,8,9,10,11]. Usually, in complex indoor optical sensing scenarios, there is a multipath effect in the transmission of optical signals. The placement of household items, object materials, spatial area, and other factors can affect the transmission of optical signals, but their monitoring data are mostly fixed and unchanged. But when users move in space, the original transmission path is disturbed, resulting in dynamic changes in optical signal data.

### 3.2. Application of Optical Sensors in the Study of Smart Home User Behavior Recognition

With the rapid development of artificial intelligence technology and the rapid growth of human–computer interaction demand, the automation and precision of user behavior recognition have become one of the research trends in smart homes. Optical sensors with a simple structure, resistance to electromagnetic interference, and a wide range of measurement objects, as key equipment for data collection and analysis in the process of user behavior recognition, have become important research objects in the field of smart homes for their selection and application. Combined with the content of the relevant literature, research on user behavior recognition is divided into six directions, and the relevant research cases illustrate the user behavior recognition technologies used more in smart homes; the main application of optical sensors is listed in Table 2.

It can be seen that in the field of smart homes, the research on user’s gesture recognition, eye movement recognition, emotion recognition and other recognition methods mainly applies optical image sensors, infrared reflection sensors and other optical sensors. They have low infringement on users, moderate installation costs and strong flexibility and have broad development prospects in user activity behavior analysis, physiological signal perception, and other aspects. In the process of user behavior perception, non-contact sensing devices and wearable sensing devices are mainly deployed to monitor and identify behavioral actions, vital signs, etc. Katsutoshi Masa et al. [21] integrated infrared light reflection sensors, IMU sensors and microcontrollers into eyewear devices, enabling them to simultaneously detect facial expressions and eye movements, thereby verifying the feasibility of developing a user-defined facial expression set. Mihai Nan et al. [25] used optical image sensors with image processing techniques to process human action behaviors in video sequences on a frame-by-frame basis. They comprehensively identify and analyze user behavior features from three aspects: joint position, bone features, and motion speed. Shota Mashiyama et al. [26] designed a fall detection system using low-resolution infrared array sensors. Monitoring the infrared radiation temperature distribution of the human body to identify the fall action makes up for the problem that the traditional image sensor is affected by dark environments, fog, etc., which make it difficult to collect images.

At the same time, as a system based on collaborative work among various devices, smart homes sometimes also assist in user behavior recognition based on environmental change monitoring. This method usually applies light intensity sensors, optical gas sensors, sound sensors, temperature sensors, etc. to build a context-centered behavioral user recognition system. By detecting environmental information such as light intensity, obstacles, volume, temperature, etc., it assists smart home systems in analyzing user behavior, which helps to better understand and explore user daily behavior and behavior patterns. Caetano Mazzoni Ranieri et al. [27] proposed a multimodal user behavior recognition framework centered on the home environment, applying deep learning algorithms to extract features from 2D CNN modules based on camera, inertial sensor, and environmental sensor data to help improve recognition accuracy. Samuel Tang et al. [28] proposed a smart home lighting system using the light sensors in smartphones to collect ambient light data. The closed-loop feedback system formed by the light sensor, lighting fixtures, and main controller improves the convenience of equipment installation and the efficiency of indoor light regulation. Kabalan Chaccour et al. [29] designed a medical walker for elderly visually impaired individuals, which uses ultrasonic sensors and infrared optical sensors for obstacle detection to help monitor stationary and moving objects around the walker, reducing the safety risk of passing through obstacles.

### 3.3. Technical Framework of Optical Sensors in Smart Home User Behavior Recognition Research

**Figure 1:**
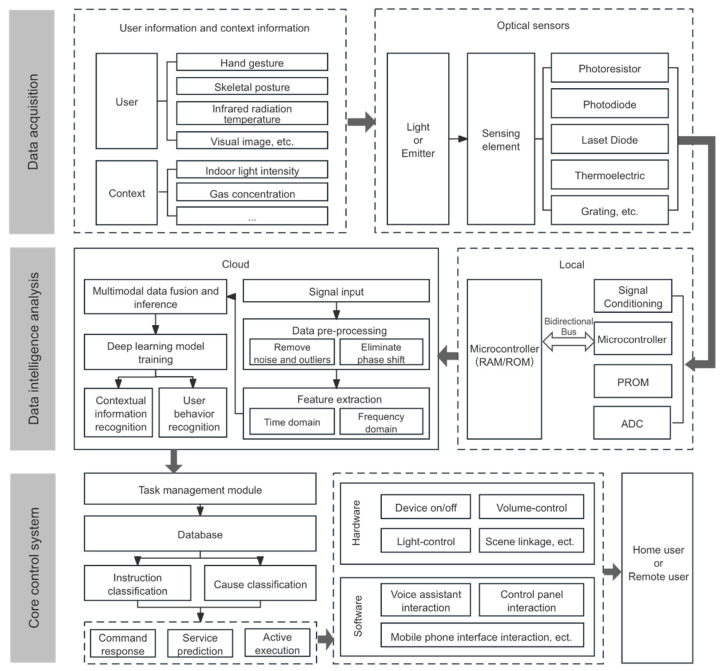
Technical framework of optical sensor-based smart home user behavior recognition.

Optical sensors have a wide range of categories and can detect a wide range of objects and have strong potential for development in smart homes. At present, in smart home systems, research on user behavior recognition is mainly carried out through a single optical sensor. Some studies have begun to combine context-aware technology and apply various sensors for environment-aided information recognition. Combining the above optical sensor working principle and relevant cases for user behavior perception and environmental information monitoring, the technical framework of optical sensors in smart home user behavior recognition research is analyzed from three modules: data collection, data intelligent analysis in the cloud, and smart home data feedback, as shown in Figure 1.

(1) Data collection module. The process primarily involves changes in user and contextual information, as well as data conversion by optical sensors. Optical sensing devices such as optical image sensors, infrared array sensors, and motion sensors collect information like user location, activity trajectory, and gestures. In addition, contextual information is gathered as a supplement, with sensors like light intensity sensors and smoke sensors collecting environmental data such as ambient light levels and smoke gas concentrations in the home. The sensing principles of components like photoresistors, laser diodes, thermoelectrics, and gratings. Some convert light signals into electrical signals, others directly detect infrared thermal radiation emitted by the human body, and some generate spectra for analysis [30,31,32].

(2) Data intelligence analysis module. The process includes two main components: local data analysis and cloud data analysis. Initially, data conversion occurs within the sensor, and bidirectional connections to the microcontroller are digital signals. Programmable read-only memory (PROM) can be used for digital compensation. Subsequently, data analysis is performed in the cloud, based on cloud computing technology and various deep learning algorithm models in computing. In data preprocessing, techniques like the moving average method, median filters, and outlier removal algorithms are employed for denoising and eliminating anomalies; methods such as phase subtraction and linear transformation are applied to correct phase shifts [33,34]. For data feature extraction, transformations are conducted using methods like fast Fourier transform, short-time Fourier transform, discrete Hilbert transform, and discrete wavelet transform. The sensors are then trained using deep learning models such as convolutional neural networks and long short-term memory networks, enabling autonomous optimization [35,36,37]. This results in the recognition of user behaviors such as location, limb posture, and gesture actions.

(3) Core control system. The primary functions are task management and service feedback. The smart home control system integrates recognized user behavior and contextual information. In the task management module, it compares this information with the database to categorize instructions and reasons, choosing to respond with command answers, service predictions, and proactive execution feedback. Considering the different interaction needs of in-home and remote users, the smart home system offers varied data feedback presentations. These include executing practical functions like controlling device on/off, volume adjustment, light adjustment, and scene linkage in hardware products. Additionally, software products provide a multimodal interactive experience for users through voice assistant interfaces, control panel interfaces, and mobile interfaces, offering visual, auditory, and tactile interactions.

Overall, light intensity sensors, image sensors, infrared sensors, laser sensors, etc., which are more frequently used in smart homes, have the advantages of low cost, small size, high sensitivity and fast processing speed. However, how to distinguish and collect various types of light signals; how to monitor and track users in real-time and accurately identify the complex behavioral information of target users; how to apply various types of sensors to improve the accuracy of user behavior analysis through multimodal contextual information and enhance the product initiative and the utility of environmental information in smart homes, is the key to the research and application of optical sensors.

## 4. Visual Analysis of User Behavior Recognition Research Based on Optical Sensors

### 4.1. Evolutionary Changes

**Figure 2:**
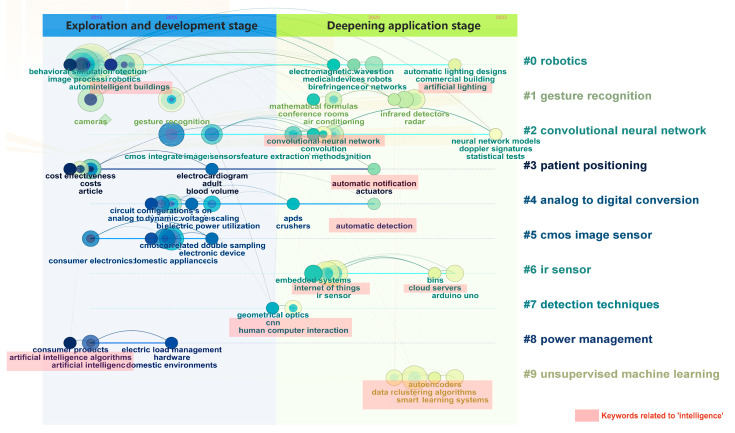
Timeline mapping.

The development of user recognition technology based on optical sensors until 2022 has already acquired the basic implementation capabilities such as user behavior data collection and information data analysis. By analyzing timeline mapping (e.g., Figure 2) and observing the process of keyword changes over time under different clustering themes, the research process for optical sensors in the field of smart home user behavior recognition during 2013–2022 can be divided into the following two stages:

(1) Exploration and development stage (2013–2017): Early research on user behavior recognition of smart homes based on optical sensors mainly focused on image sensing technology, which compares and identifies user behavior information with defined body shape specification, gesture norms, etc., but there are limitations in recognizing complex user behavior. At the same time, this stage began to apply big data, artificial intelligence algorithms, etc., to optimize the research on optical sensors for intelligence, low power consumption and high efficiency. (2) Deepening application stage (2018–2022): User behavior recognition based on optical sensors is more diversified, deep learning technology is maturely applied in the smart home field, and the ability of data processing and analysis is continuously innovated and improved, making user recognition technology more accurate and intelligent. At the same time, attention is being paid to user experience. The researchers distinguish user needs from different dimensions such as elderly users and patient users, and focus on the naturalness of the identification process, the privacy and security of data analysis, and the timeliness of information feedback, etc.

### 4.2. Research Hotspots

**Figure 3:**
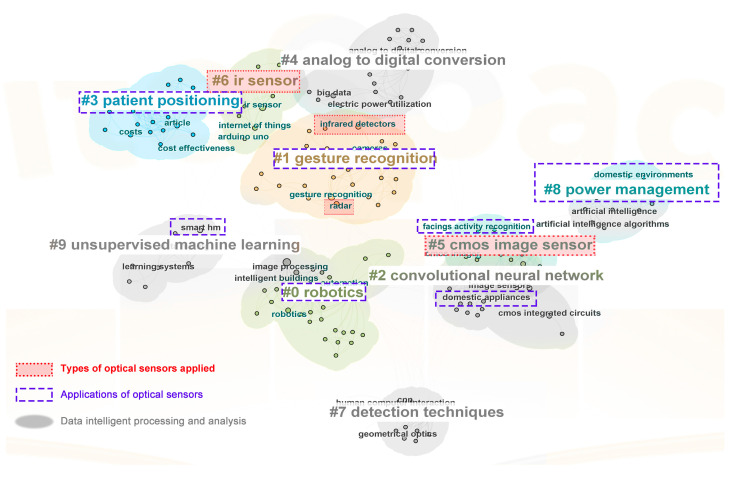
Types, applications and data analysis of optical sensors.

Figure 3 shows the keyword co-occurrence and clustering diagram. The keywords are classified and analyzed from three aspects: types of optical sensors, application fields and data analysis and processing. It is found that there are various types of optical sensors and optical sensing methods such as image and infrared that are mainly applied to research on gesture recognition, facial recognition and daily behavior recognition in the field of smart home user behavior recognition, with good application prospects. The data fusion analysis of multiple optical sensors also helps to improve the accuracy and efficiency of user behavior recognition. Along with the continuous maturity of the Internet of Things, artificial intelligence, big data and other technologies, the intelligent innovation and development of optical sensors have been promoted. Based on cloud computing technology, deep learning algorithms such as convolutional neural networks and recurrent neural networks are applied to realize intelligent processing and the analysis of optical sensor data, which can effectively improve the data processing and computing ability, model training ability, hard disk storage ability and information transmission speed of optical sensors [24]. At the same time, the security and privacy of smart homes, as the user needs, are key to improving the user experience. The issues of installation costs, energy consumption, and other expenses are also fields for researchers to explore.

Through the interpretation and analysis of various keywords, combined with the above-mentioned development background of optical sensor-based smart home user behavior recognition research, the following three research hotspots are summarized: (1) Comprehensively apply various optical sensors to design diversified, ubiquitous and systematic user behavior recognition technologies and more accurately understand user intentions through diverse and heterogeneous sensor data. (2) Develop and apply machine learning algorithms to continuously improve the performance of optical sensors, mitigate the impact of environmental changes on data, and achieve higher recognition accuracy and more intelligent data analysis levels. (3) Strengthen the awareness of privacy protection, install and use various optical sensing devices reasonably, with a view to adapting to different user groups and reducing the sense of intrusion brought by the devices.

### 4.3. Future Research Trends

Guided by the development of artificial intelligence technology, we explore the intelligent and innovative research of optical sensors based on new materials, new principles and new algorithms. Through reviewing and analyzing the relevant literature and application cases, it has been found that optical sensors, with advantages such as wide recognition range and natural interaction, have a wide range of applications in smart homes, especially in behavior recognition for special populations such as the elderly and visually impaired. At present, there are many types of sensing devices, which are prone to problems such as occupying space, disorderly placement, and confusion among users in home environments. In the process of using optical sensors for special populations, there are also higher requirements for ease of use, recognition accuracy, safety, etc. [38,39]. At the same time, optical sensors are affected by ambient light, ambient temperature, obstacle occlusion, etc. in the process of optical signal acquisition, and the optical signals emitted by different sensors may interfere with each other [40].

To solve the above problems, the future research trends of optical sensors in smart home user behavior recognition are summarized by combining the literature review research content, mainly including the following points:

(1) The approach involves integration and multimodality. By analyzing the needs of optical sensors based on different functional scenarios, the strategy involves integrating devices like image sensors and infrared sensors to reduce equipment size and enrich recognition capabilities. The sensors are designed with aesthetic appeal and modular structures, aiding users in distinguishing sensor functions and enhancing installation convenience. In complex user environments, sensors of various modalities are distributed and assembled, merging data from optical sensors with other types (such as acoustic and electromagnetic wave sensors). This integration of diverse and heterogeneous sensor data aids in understanding user intentions and environmental conditions. The complementary data from different sensors effectively identify the actions or areas of targeted users and comprehensively analyze information characteristics like user emotions and preferences. This enhances recognition efficiency and detection precision, allowing the home system to offer more accurate intelligent services.

(2) Adaptive Control and Optimization. By utilizing deep learning algorithms like artificial neural networks and convolutional neural networks, adaptive control capabilities are continually enhanced. This is beneficial for the smart home system to dynamically adjust device operations based on multimodal sensor data, enabling more targeted recognition of user behaviors and activity areas in different scenarios, and offering stronger adaptability for diverse user groups. Combined with contextual awareness technologies, the optical sensors’ environmental adaptability and user tracking monitoring are enhanced. A key research focus should be on how to actively learn and adapt to user behavior based on context-aware user behavior models, utilizing methods like deep learning and semantic analysis [41].

(3) Contextualization and Strong Integration. In the smart home service feedback process, new display technologies, AI image generation, and mobile connectivity integration enrich the interactive experience of home appliance interfaces. By combining contextual awareness and deep learning technologies to detect and analyze changes in user, environment, and device information, the system can more accurately understand user intentions and contexts. This improves the personalization of interactions, proactivity of services, and practicality in various environments within the smart home. The design of a multi-channel integrated interactive experience provides users with appropriate visual, auditory, and tactile feedback, emphasizing the naturalness of the interaction process. Simultaneously, intelligent product functions are linked according to user needs in different scenarios, achieving intelligent contextual recognition. This approach helps ensure the coherence and consistency of the interactive experience.

(4) Privacy Protection and Communication Security. Smart home systems rely on sensors for data collection, and optical sensors, due to their non-intrusive nature and high precision, may be more covert than traditional cameras in collecting home environment data. They hold potential to minimize disruption to residents’ daily lives and infringement on their privacy [42]. Simultaneously, developing more secure communication protocols and encryption technologies is crucial. This ensures the safe and intact transmission of data exchanged between optical sensors, other devices, and the central processing unit. Researching how to process and store data collected by optical sensors locally, reducing reliance on cloud storage, is vital for preventing external attacks and data breaches.

To sum up, the research in smart home user behavior recognition based on optical sensors has gradually moved from the most basic image sensing recognition to the intelligent analysis of multi-sensing data. In the future, in terms of information collection, in addition to targeted extraction and analysis of user behavior information, it is also necessary to focus on the contextual information in which the user is located, so that the smart home system can understand the user scene in a timely manner and support more natural human–computer interaction methods such as active interaction and implicit interaction. In terms of optical sensing technology, it is necessary to continuously innovate the identification methods and integrate computer technology and communication technology, in order to effectively help realize the non-invasive and proactive intelligent state of home products.

## 5. Application Scenarios of Smart Home User Behavior Recognition Based on Optical Sensors

### 5.1. Application Scenarios—Taking Elderly Users’ Fall Monitoring Situation as an Example

**Figure 4:**
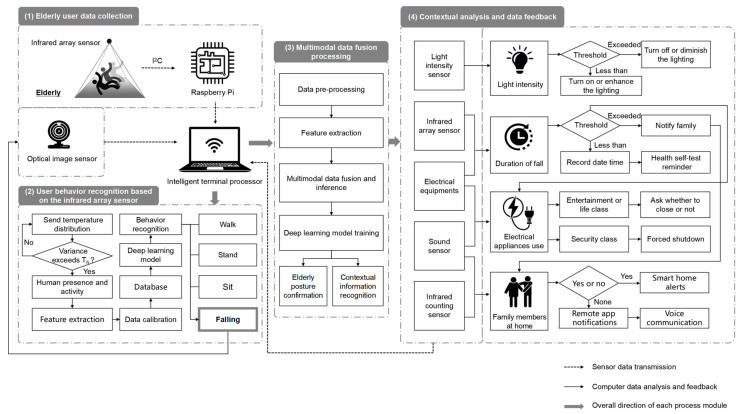
Technical framework of smart home data collection and processing based on fall monitoring scenario for the elderly.

As one of the core users of smart homes, the comfort, convenience, and safety of home life experience for the elderly population is the focus of several researchers. We take the elderly user fall monitoring scenario as an example and build a detailed technical framework for smart home data collection and processing based on the above case study and the literature visualization analysis content [43,44]. The aim is to illustrate the feasibility of research trends in practical application scenarios through practical case studies, so as to solve some problems in the application of optical sensors in smart home user behavior recognition. The relevant technical framework mainly includes four stages: data collection from elderly users, user behavior recognition based on an infrared array sensor, multimodal data fusion processing, and contextual analysis and data feedback, as shown in Figure 4.

(1) Elderly user data collection: The smart home system mainly uses infrared array sensor data to monitor the infrared radiation temperature of the human body, so as to identify fall action through infrared temperature distribution profiles. When the system recognizes an elderly person falling, it then opens an optical image sensor for status confirmation and video recording, jointly monitoring user data, providing more comprehensive user data for the cloud-based intelligent processor, and ensuring the accuracy of elderly user falling behavior monitoring.

(2) User behavior recognition based on the infrared array sensor: Firstly, it is determined whether the user is active or not by analyzing the temperature distribution data of the detection area. Secondly, feature extraction is performed and the data calibrated according to the installation height and angle of the sensor. Finally, the user behavior is identified by matching relevant behavior categories in the database.

(3) Multimodal data fusion processing: Machine learning algorithms are applied to process multimodal sensing data received by terminal processors. By combining the data results from optical image sensors, it is accurately confirmed that the elderly person is in a falling posture. In the process of context analysis, through the fusion and inference of various sensor data, as well as the use of cloud computing for large-scale, high-volume sample collection and training, it is possible to achieve data sharing and collaborative work and achieve the effect of adaptive identification of different situational information, helping smart homes to better provide diversified and personalized automatic feedback services.

(4) Contextual analysis and data feedback: A light intensity sensor may be applied to identify the change in light and darkness in the room, so that the smart home can automatically adjust lighting systems and assist optical image sensors in recording user behavior. Meanwhile, the smart home system can also analyze and encode information data based on other contextual information, such as appliance usage, fall duration, family members at home, and ambient temperature. Through the cloud-based intelligent processor, applying cloud computing technology, it can monitor the living conditions of the elderly in real time, such as activity, sleep, heart rate, etc. Combined with the user needs to set the appropriate feedback mode and form, smart homes can respond to a user’s intentions and environmental changes.

### 5.2. Future Prospects

The construction of the smart home technology framework centered on elderly users, taking into account the user’s awareness of privacy and security, is mainly based on the infrared array sensor for user fall behavior monitoring and the application of other sensing devices for auxiliary information identification. After accurately collecting and identifying user and contextual data, functional feedback is provided through smart home products that combine software and hardware to solve the problems of unawareness and the inability of elderly users to call for help after falling at home. It provides a certain idea expansion and reference for the interaction design field to study how to reasonably use various sensing devices in the home and use intelligent technology to ensure the fluency of interaction between elderly users and smart homes.

The next step involves the iterative design of a smart home user behavior recognition framework centered on optical sensors, particularly for monitoring elderly falls. The primary research and problem-solving areas include: (1) Tracking and monitoring the behavior of the main target user using optical sensors in a multi-person household environment. (2) Differentiating and processing various types of light signals in the integration of optical sensors. (3) Exploring and developing more user scenarios, considering different users’ preferences and requirements for smart product functionalities, and enhancing the machine learning capabilities of artificial intelligence systems. (4) Building a multimodal sensor network centered on optical, acoustic, and electromagnetic wave sensors within the smart home environment, rich in information modalities, to achieve accurate identification and input of user information.

## 6. Conclusions

This paper mainly studies the user behavior recognition of smart homes based on cloud computing technology, focusing on the field of optical sensors. Through case analysis and a literature review, we were able to deeply understand the advantages and shortcomings of optical sensors in user behavior recognition research, explore its research hotspots and development trends, and propose innovative research on optical sensors in the field of smart homes from the perspectives of sensor integration technology, multimodal intelligent sensing technology, and enhanced artificial intelligence technology. At the same time, we combined human–computer interaction and context-aware technology to explore a new model of user behavior recognition in smart homes. Taking the elderly fall monitoring scenario as an example, through the use of cloud computing technology, we proposed a framework of data collection and processing technology for smart homes based on optical sensors through practical application scenarios, providing new ideas for the design of age-friendly smart homes.

This study summarizes and analyzes the arguments based on the scientific and objective literature, and provides a rich, detailed and convincing theoretical basis and factual foundation for the study of optical sensors in the field of user behavior recognition for smart homes. Meanwhile, combined with a user-centered interactive design, it inspires researchers to investigate technical improvements and innovative applications of optical sensors from the perspective of promoting and improving user experience, which helps in exploring more application scenarios for optical sensors in smart homes and building a more active, natural and personalized home environment.

## Figures and Tables

**Table 1:** Types of optical sensors commonly used in smart homes.

Types of Optical Sensors Commonly Used in Smart Homes	Working Principle	Transmission Structure	Scenario
Optical image sensor	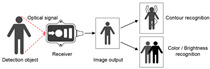	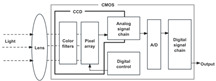	Video calling, security surveillance, gesture recognition, facial recognition, etc.
Passive infrared sensor	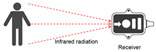	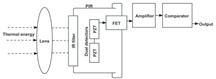	Automatic doors, automatic faucets, motion sensor lights, intrusion alarms, etc.
Active infrared sensor	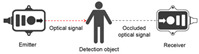	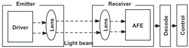	Remote control of household appliances, anti-theft doors and windows, etc.
	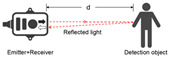	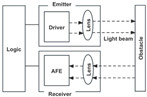	Human monitoring, smoke detection, facial expression recognition, etc.

**Table 2:** Application of optical sensors in the study of smart home user behavior recognition.

Topic	Sub-Research Direction	Related Research Cases	Optical Sensors
Application of optical sensors in the study of smart home user behavior recognition	Multimodal Behavior Recognition	Multimodal sensing data integration based on deep learning [12]	Optical image sensors, infrared sensors, etc.
	Gesture Recognition	Fingertip monitoring [13]. Gesture trajectory recognition [14]. Fingerprint recognition [15].	Optical image sensor, polarimetric fiber sensor, etc.
	Speech Recognition	Personalized speech recognition system [16]. Noise cancellation [17]. Conversational context awareness [18].	None
	Eye Tracking	Gaze dwell time monitoring [19]. Sleep status monitoring [20].	Infrared oculography (IROG), electro-oculography (EOG), video-oculography (VOG), etc.
	Emotion Recognition	Facial expression recognition [21]. Human movement and gesture monitoring [22]. Multimodal emotion recognition [23].	Optical image sensor, infrared photo-reflective sensor, etc.
	Activity Recognition	Human indoor positioning [24]. Human skeletal pose [25]. Human infrared radiation temperature distribution [26].	Optical image sensor, infrared proximity sensor (IRPS), infrared array sensor, etc.

## Data Availability

The datasets used and/or analyzed during the current study are available from the corresponding author on reasonable request.
